# Using social network analysis to understand online Problem-Based Learning and predict performance

**DOI:** 10.1371/journal.pone.0203590

**Published:** 2018-09-20

**Authors:** Mohammed Saqr, Uno Fors, Jalal Nouri

**Affiliations:** Department of Computer and System Sciences (DSV), Stockholm University, Kista, Stockholm, Sweden; University of Zurich, SWITZERLAND

## Abstract

Social network analysis (SNA) may be of significant value in studying online collaborative learning. SNA can enhance our understanding of the collaborative process, predict the under-achievers by means of learning analytics, and uncover the role dynamics of learners and teachers alike. As such, it constitutes an obvious opportunity to improve learning, inform teachers and stakeholders. Besides, it can facilitate data-driven support services for students. This study included four courses at Qassim University. Online interaction data were collected and processed following a standard data mining technique. The SNA parameters relevant to knowledge sharing and construction were calculated on the individual and the group level. The analysis included quantitative network analysis and visualization, correlation tests as well as predictive and explanatory regression models. Our results showed a consistent moderate to strong positive correlation between performance, interaction parameters and students’ centrality measures across all the studied courses, regardless of the subject matter. In each of the studied courses, students with stronger ties to prominent peers (better social capital) in small interactive and cohesive groups tended to do better. The results of correlation tests were confirmed using regression tests, which were validated using a next year dataset. Using SNA indicators, we were able to classify students according to achievement with high accuracy (93.3%). This demonstrates the possibility of using interaction data to predict underachievers with reasonable reliability, which is an obvious opportunity for intervention and support.

## Introduction

Problem-Based Learning (PBL) is a constructive self-directed and collaborative approach to learning. The underpinning philosophy behind PBL is that learning occurs as a result of active co-construction of meaning, dialogue, and negotiation with peers. Learning is typically motivated by using challenging, authentic real-life problems [1–4]. The main three features of PBL are a *problem* as a trigger for learning, a *facilitator* commonly known as the tutor, and small group *collaborative interaction* [5–7]. The process is supposed to help the student to activate prior knowledge as well as to elaborate through discussion with peers, explain to self and others, and answer queries. Elaboration is expected to promote cognitive and motivational self-regulation and enhance life-long learning skills [3–5].

With the emergence of Internet and Computer Supported Collaborative Learning (CSCL), several institutions have embraced a blended PBL approach (using CSCL or wikis to support face-to-face PBL) [8–11]. The blended approach harnesses the possible benefits of learning through, for instance, asynchronous communication and permanent access to content [8, 11, 12].

Applying the constructivist model to explain learning in PBL, three factors are often recognized. First, student factors such as interest in subject matter and prior knowledge. Second, tutor factors such as knowledge of subject matter, scaffolding and effective group facilitation and, third, content factors such as the quality of the problem [3, 6, 13–15]. The interaction of these factors, as well as the social and cognitive interaction, are thought to be the mechanism of learning in PBL [3, 6]. Interaction in online learning can be bidirectional in three forms, learner-teacher, learner-learner, and learner or teacher-content [16–18].

The value of interactivity in technology-enhanced learning has long been emphasized as an essential constituent of the learning process [16, 18–20]. Besides, it is supported by evidence from large-scale systemic reviews and meta-analyses. For example, Bernard et al. [21] concluded that increasing interaction among learners, teacher, or content positively enhances learning (average effect of 0.38). In a meta-analysis by Borokhovski et al. [22], courses that promote student-student interaction were found to enhance learning significantly.

Interactions in online problem solving require learners to engage in two types of dialogical aspects. The first is the *content aspects* (interactions related to the subject of the problem in the discussion) and the second is the *relational aspects* (interactions related to communicative activities) [23, 24]. Effective interactions in the relational space is a necessary precondition for successful problem discussion and the realization of the goals of problem-based learning [23, 24]. According to Azer et al. [17] who recently reviewed group interaction in PBL, there are deficiencies and gaps in the knowledge available regarding the impact of group interactions on student’s learning. The vast majority of research on interaction in PBL have focused on studying the content dimension through qualitative methods, such as content analysis, interviews, and text mining, or indirect examination and exploration by means of surveys or open-ended questionnaires [17]. The relational aspects of PBL remain largely unstudied and little is known about the value of studying the relational aspects of online PBL by novel techniques such as Social Network Analysis (SNA). By using SNA and learning analytics to study students’ positions, relations, and interactions, we might enhance our understanding of online behavior, tracking engagement and academic achievement [25–30].

Learning analytics seem to have the potential to assist educators to early identify underachievers and possibly shed light on the factors that might help improve their engagement and improve attrition rates [25, 26, 31, 32]. Underachieving students who are at risk of failing a course or dropping out from a program is a noteworthy problem that incurs a considerable cost at many levels. Albeit the magnitude of the problem seems to be substantial, it is still poorly studied. Therefore, the preventive mechanisms are either suboptimal or poorly implemented [33].

Although studies using learning analytics and SNA to investigate the participation in online discussions are few, initial results are encouraging. For instance, Romero et al. [34] reported a positive correlation between in-degree (number of received interactions) and degree centralities (total number of interactions) and the possibility of passing a course. Likewise, Hommes et al. [35] found that degree centrality to be strongly correlated with students’ learning; the correlation was more substantial than academic motivation, prior performance, and social integration. Similar results were reported by Joksimović et al. [36] who found weighted degree centrality (total number of interactions accounting for importance) to be the most significant factor for predicting student performance. Other researchers found that the student’s social capital (strength of personal networks) is correlated with higher academic achievement [37, 38]. However, such results have not been replicated, and contradictory findings have been reported [30, 36, 39, 40]. Studies that investigated SNA parameters in multiple courses have faced the same reproducibility problem. For instance, Ángel et al. [30] obtained inconsistent results from a course to the other. In some courses, there was no correlation with performance, while in others, the correlation was positive and significant. The authors called for investigating the context in which SNA can be reliable predictors of performance.

Despite the challenges mentioned, SNA may be principally effective in studying the relational dimension of blended PBL by means of visual analytics and quantitative mathematical analysis [26, 27, 29, 30, 41]. With the support of visual analytics the PBL group structure, the learner-learner, and the learner-tutor interactions can be mapped in order to identify influential and isolated learners as well as group functioning [27, 28, 42]. Furthermore, SNA quantitative network analysis can be used to estimate the power of each collaborator, the strength of the relationships and the overall group properties [42–44]. As such, SNA quantitative network analysis may be of particular significance in studying social interactions in online PBL, and how they relate to achievement and the PBL process. Our review of the literature leads us to conclude that the value of SNA measures for predicting performance using learning analytics techniques is an uncharted territory of inquiry in the field of online PBL.

Therefore, we argue here that using SNA to study online PBL interactions might offer insights on multiple levels that help us to predict under-achievers and uncover the significance of the role of learner-learner and learner-tutor interactions.

The general research question of this study is: *How can SNA contribute to our understanding and enhancement of the online PBL process*? This general research question is divided into the following sub-questions:

- RQ1: How do social network analysis indicators correlate to performance (in terms of grades) in online PBL?
- RQ2: How far can SNA indicators be used as reliable predictors of performance in online PBL?

## Methods

### The context

**Fig 1:**
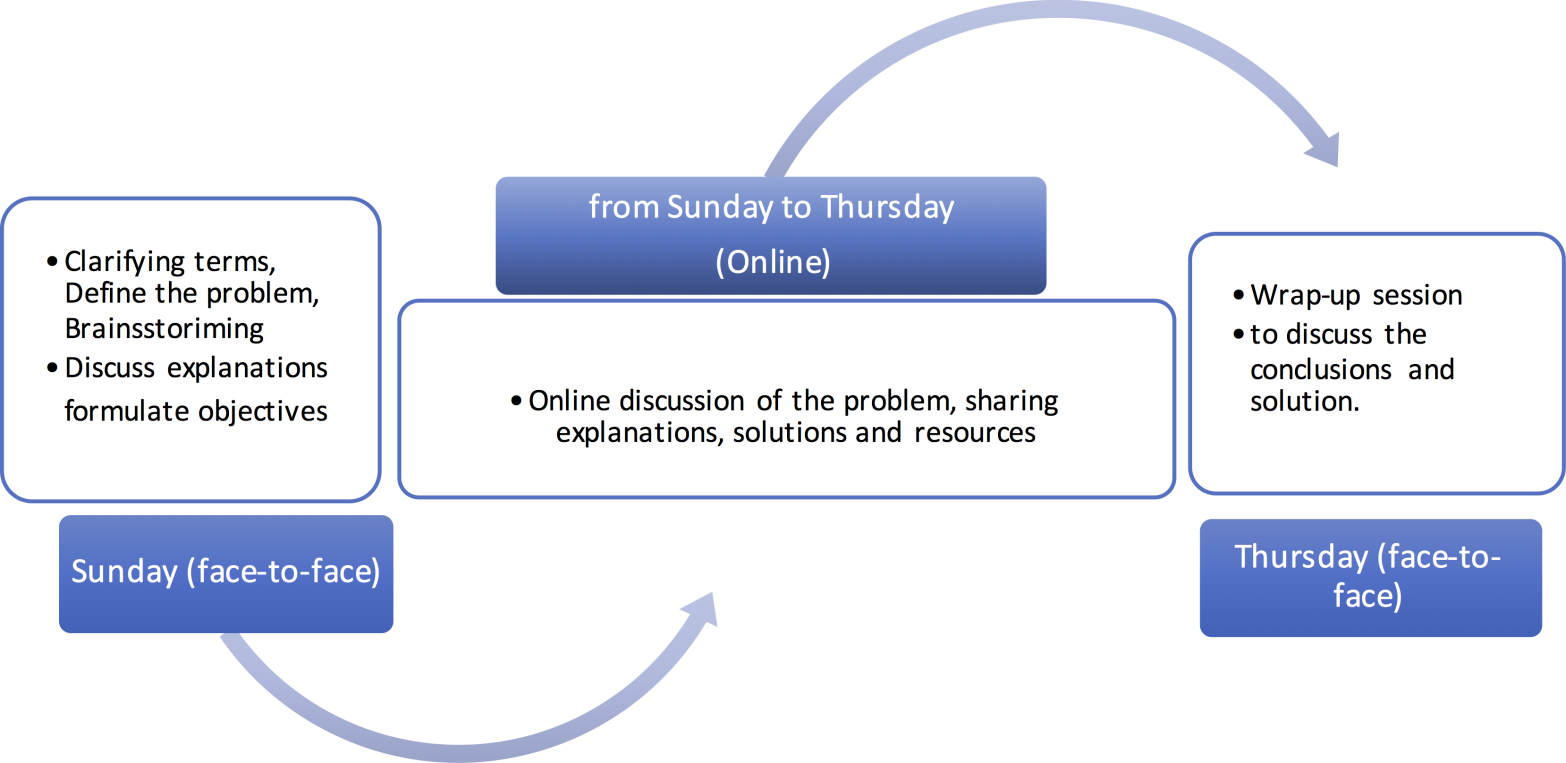
The typical stages of the BPBL process. A face-to-face session followed by long online discussion throughout the week followed by a wrap-up session at the end of week.

The study included four courses in the College of Dentistry, Qassim University, Saudi Arabia, namely: Body Systems in Health and Disease (QDENT 211), General Surgery (QDENT 212), Neuroscience (QDENT 213), and Principles of Dental Sciences (QDENT 214). These are all the courses of the second year that has blended PBL (BPBL) as a teaching method. As outlined in Fig 1, the typical BPBL is divided into two face-to-face sessions. During the first session the students discuss the problem, suggest explanation and formulate learning objectives to be learned. Then online discussions continue throughout the week to discuss the learning objectives identified earlier, share learning resources, concept maps, and explanations. By the end of the week, students are expected to demonstrate their learning and discuss conclusions [7, 45], an illustration of the process is outlined in Fig 1. The college started to implement blended problem-based learning in 2009 [45]. An evaluation of the approach concluded that it was well received by students and moderators as the approach helped enhance interactivity and encouraged participation [8, 46].

### Data collection and analysis

The process of data collection and interpretation in this research followed the standard data mining process as described by Romero et al. [44, 47], which can be divided into the following steps:

**A. Data collection:** The level of analysis in this study required collection of metadata about the attributes of individual users, groups, and courses as well as the properties of each post. Interaction data were extracted from Moodle database using Structured Query Language (SQL) custom queries. Using SQL database queries for data gathering is more flexible, and enables detailed information analysis compared to using Moodle logs [47].

The extracted data included user information (online user ID, course ID, group ID, course title and user email) and post information (post ID, post subject, post content, parent forum, post author, replies, author of the reply, post time, course, and group ID). Performance data were obtained from final course records.

**B. Data preprocessing:** users’ records were cleaned (3 corrupted records were removed), data from different sources were combined in a single master sheet. Personal information was anonymized and coded to remain private. The data were converted to a format compatible with the analysis tool *Gephi*. Each BPBL group were processed in a separate network file since group discussions were separated from each other online. Course networks were also studied separately to account for all interactions in the course beyond BPBL.

**C. Data Analysis and Interpretation:** To have a general overview and summary of the dataset, we performed descriptive statistics of courses, groups, and interactions. Both visual and mathematical analysis of social network were performed. SNA visualization was performed to explore the social structure in each course and group and to guide the analysis. SNA visualization has a powerful summarizing function of interactions among participants and the communities they are members of (courses and groups in this context). It also facilitates the interpretation of quantitative network analysis. Quantitative network analysis was performed to calculate the social network parameters for each course, group and the centrality scores of each student for descriptive statistics and to serve as features for further inferential analysis and predictive modeling. To answer the first research question, the correlation among social network parameters and student’s performance was calculated using the Spearman correlation coefficient.

To answer the second research question about how far SNA indicators can be used as reliable predictors of performance in online PBL, two types of predictive models were used. The first type (explanatory model) used statistical modeling to build and test a hypothesis; in this model, the factors thought to influence the outcome in the PBL process were included, which are the student, the tutor, and the group [3, 13, 14]. The goal of the explanatory model was to investigate if SNA could capture the interactivity and relational construct of PBL, and as a theory based predictive learning analytics model. The second type was predictive modeling, in which the objective was to use the available data to investigate the possibility of forecasting future students’ performance. The goal was to compare the theory-driven approach to a non-theory driven approach, and use modern machine learning methods for validating the reliability of the resulting model. Furthermore, predictive models test the possibility of predicting a future event, as such, demonstrate the potential of early intervention. To validate the results, a next year data-set of the same four courses were used. For an in-depth review of the predictive models in education, please refer to reference [48, 49].

#### Descriptive statistics

We calculated each course and group size, total number and type and of interactions in each BPBL group and course separately. Interactions were sub-classified according to source and target as Student-Student (S-S), Student-Tutor (S-T), and Tutor-Student (T-S). Additionally, SNA parameters of each course and PBL group were calculated.

#### Social network analysis

The open-source SNA software Gephi (version 9.1) was used for network visualization and analysis. Gephi is a powerful interactive open-source SNA application, commonly used for network visualization and exploration with advanced features such as filtering, clustering, and partitioning capabilities [50]. Two types of analyses were made:

#### 1. Visualization

A social network has two elements, the network actors (nodes) and the ties (edges) connecting them. In Blended PBL context, students and tutors represent the nodes, and the interactions represent the edges. Social networks are visually represented by mapping interactions (edges) among the actors (nodes) in a graph known as a “sociogram” [43]. The sociograms were rendered using the *Fruchterman Reingold algorithm*, a widely used force-directed layout algorithm that uses physical simulation to draw each node according to connected edges; the resulting visualizations are easy to interpret and understand with fewer edge crossings [51]. *Fruchterman Reingold algorithm* rendered sociograms in a circular manner and was recognized as being useful in demonstrating the relationship between learners and instructors [30]. Visualization of the interactions was done to have an idea about the overall interactions in each group, the relationship between participants and to possibly discover the position and significance of each role, which in turn, would help interpret the quantitative parameters correctly.

#### 2. Quantitative network analysis

Network quantitative analysis is a mathematical approach to quantify the prominence of users and the value of connections in a social network. The prominence of individual users is usually expressed as centrality measures, prominence can be expressed differently according to the perspective and the construct measured. The emphasis in this study was on the centrality measures that represent interactivity, knowledge sharing and discussion [52, 53]. The main constructs were the quantity of participation, the role of mediation and brokerage of knowledge transfer in the group, the strength of connectedness and group cohesion, relationship to group members, and importance of neighbors (social capital). Three sets of parameters were calculated, individual user parameters, BPBL group parameters, and course parameters. The following parameters were calculated for each student.

**The quantity of participation parameters**:

- In-degree centrality: also, known as prestige, is the total number of interactions (edges) received by a user. It is an indication of influence and authority [54].
- Out-degree centrality: the total number of interactions posted by the user, it is a quantification of the activity in the network, the higher the out-degree centrality, the more active is the user [54].
- Degree centrality is the sum of outgoing (Out-degree) and incoming (In-degree) interactions [54, 55].

**Position in information exchange**

- Betweenness centrality measures the number of times a user played a role in mediating information exchange or brokered the communication in a network [54].
- Information centrality measures the role of the user in the flow of information in the discussions. The higher the value of information centrality, the more influential the user in the information exchange [56].
- Closeness centrality measures how near (close) a user is to all other participants in the network. Close users are easy to reach and interact with most participants and [54, 55].

**Connectedness**

- Eigenvector centrality measures the prominence of a user considering his neighbors, a user connected to prominent users in the network will have higher values of Eigenvector centrality [54].
- Eccentricity measures the distance of a user from the further users in the network and can be viewed as an indication of a difficulty to reach or isolation [38].
- Clustering coefficient measures the tendency of a user to group (cluster) with others in the network, the higher the clustering coefficient, the more that user has communicated with more members of his group and is considered to be an indicator of group cohesion. [54, 57].
- Prestige measures:
  ○ In-degree prestige is the number of users who are directly connected to the user and can be viewed as an estimate of the size of the ego network.
  ○ Proximity prestige is the number of users who are directly or indirectly connected to the user, a measure of the range of influence.
  ○ Rank prestige is the number of connected users taking into consideration their prominence, a measure of the prominence of ego network.
  ○ Domain prestige is the number of users who are pointing to the user, a measure of influence as voted by neighbors.
- For each BPBL group network, we calculated the network size (number of nodes), density (ratio of actual to possible edges among nodes in the group), average degree (the mean degree of all nodes in the group), and average clustering coefficient (the average clustering coefficient of all nodes in the network).
- For each course network, we calculated network size, density, average degree, and average clustering coefficient.
- Final course grades were used as a measure of achievement. Students were ranked and classified. The bottom 1/3 was classified as low achievers and the top 1/3 as high achievers.

#### Statistical analysis

**RQ1**: SPSS software version 24 for Windows was used for statistical analysis. Pearson’s correlation test was performed to measure the direction and strength of correlation between variables.

**RQ2**: Stepwise backward multivariable linear regression was performed using SPSS to assess which of the interaction parameters might explain the variance in the final grade. To avoid multicollinearity, we removed correlated parameters that measure closely interrelated constructs, such as the number of interactions, number of S-S interactions, average group degree centrality, average course degree centrality, course and group density. In this case, we included only *group density* since it captured the interactivity construct, is not dependent on group size and was the variable that most correlated with performance. A correlation matrix was constructed, and predictors with a correlation coefficient of more than 0.7 were removed. Predictors that had a Variance Inflation Factor (VIF) of more than 10 or Tolerance less than 0.1 were considered for removal.

- For the categorical classification of students according to performance, we used Logistic Regression (LR). LR is a powerful predictive model, commonly used for the prediction of binary outcomes such as high versus low achievement. The Logistic Regression operator of Rapidminer studio version 7.5 was used for the prediction and validation of under-achievers.
- The following parameters were calculated to evaluate the predictive accuracy of the classification algorithms:
  ○ **Accuracy:** the percentage of correctly classified students.
  ○ **Recall** (sensitivity): is the percentage of successfully classified positive predictions divided by the total number of all positive values (True Positive Rate).
  ○ **Precision**: is the percentage of successfully classified positive predictions divided by the total number of all positive predicted values (Positive predictive value).
  ○ **F-measure**: is the harmonic mean of both the precision and the recall.
  ○ **Receiver Operating Characteristic (ROC):** is a plot of the True Positive Rate (Recall) of a model against the False Positive Rate (1 –specificity.). The area under the curve (AUC) is considered an estimation of the model accuracy, where 1.0 represents a perfect model, and 0.5 means an insignificant model[58, 59].

### Research ethics

The study was approved by the Regional Research Ethics Committee of Qassim Region after reviewing the study protocol, consent documents and the consent procedure and issued an approval of the study. An online privacy policy that details possible use of data for research and user protection guarantees was signed by all participants (reviewed by the ethical committee). Data utilized in this study were anonymized, and personal information was removed. College Privacy guidelines and policies for dealing with students’ data were strictly followed, and data collection complied with the Moodle terms of service. It is also important to mention that all students were enrolled in the course and were able to complete it regardless of signing the agreement and were able to opt out of participation in this research. The researchers of this study did not participate in teaching or grading the studied courses.

## Results

### Descriptive statistics

The study included 215 students and 20 tutors in 4 courses; each course had 5 BPBL groups, group size ranged from 10–14 students and one tutor. The total number of interactions in all courses was 6439, the highest number of interactions was 3134 in QDENT 211. Most of the interactions were among students (range 88.18% to 92.20% of all course interactions), followed by the tutor to student (range 5.91% to 8.93%). Student to tutor interactions were very few, the highest percentage was in QDENT 214, making only 2.89% of all interactions in the course, detailed statistics of each type of interactions and the distribution in each course are presented in Table 1, and Table 2 shows statistics of group interactions.

**Table 1:** Distribution and type of interactions in each course.

Type of interaction	Number of interactions	Percentage
**QDENT 214 (N 54)**		
Student to Student	1067	88.2
Student to Tutor	35	2.9
Tutor to Student	108	8.9
**Total**	**1210**	**100.0**
**QDENT 213 (N 53)**		
Student to Student	920	89.1
Student to Tutor	27	2.6
Tutor to Student	86	8.3
**Total**	**1033**	**100.0**
**QDENT 212 (N 54)**		
Student to Student	1029	92.2
Student to Tutor	21	1.9
Tutor to Student	66	5.9
Total	1116	100.0
**QDENT 211 (N 54)**		
Student to Student	2795	89.2
Student to Tutor	83	2.7
Tutor to Student	256	8.1
**Total**	**3134**	**100.0**

Group sizes ranged from 10–14, the average mean grade ranged from 68 to 95.3. Students were generally more active in the BPBL groups, therefore, the average (Av) mean *degree* of tutors was 38.61±28.52 compared to 56.04±35.88 of students, average *S-S interactions* were far higher than *T-S* (290.55 compared to 25.05). The mean *density* was 2.68±1.81, indicating that most groups showed a considerable amount of interactivity, as density values higher than one means that all group members interacted with each other. For detailed statistics of group properties, please refer to Table 2.

**Table 2:** Group descriptive statistics and network parameters.

Group	No. of S	Av grade	Av Clustering	T degree	Density	Av Degree	S to S	S to T	T to S	No. interactions
**Minimum**	10	68	0.28	5	0.53	11.85	61	0	4	77
**Maximum**	14	95.3	0.88	115	7.28	139.17	799	30	77	827
**Mean**	11.80	83.7	0.66	38.61	2.68	56.04	290.55	8.30	25.05	324.30
**SD**	1.15	8.1	0.17	28.52	1.81	35.88	191.42	8.09	21.27	203.52

Av = Average, T = Tutor, S = student.

### Visualization of course interactions

**Fig 2:**
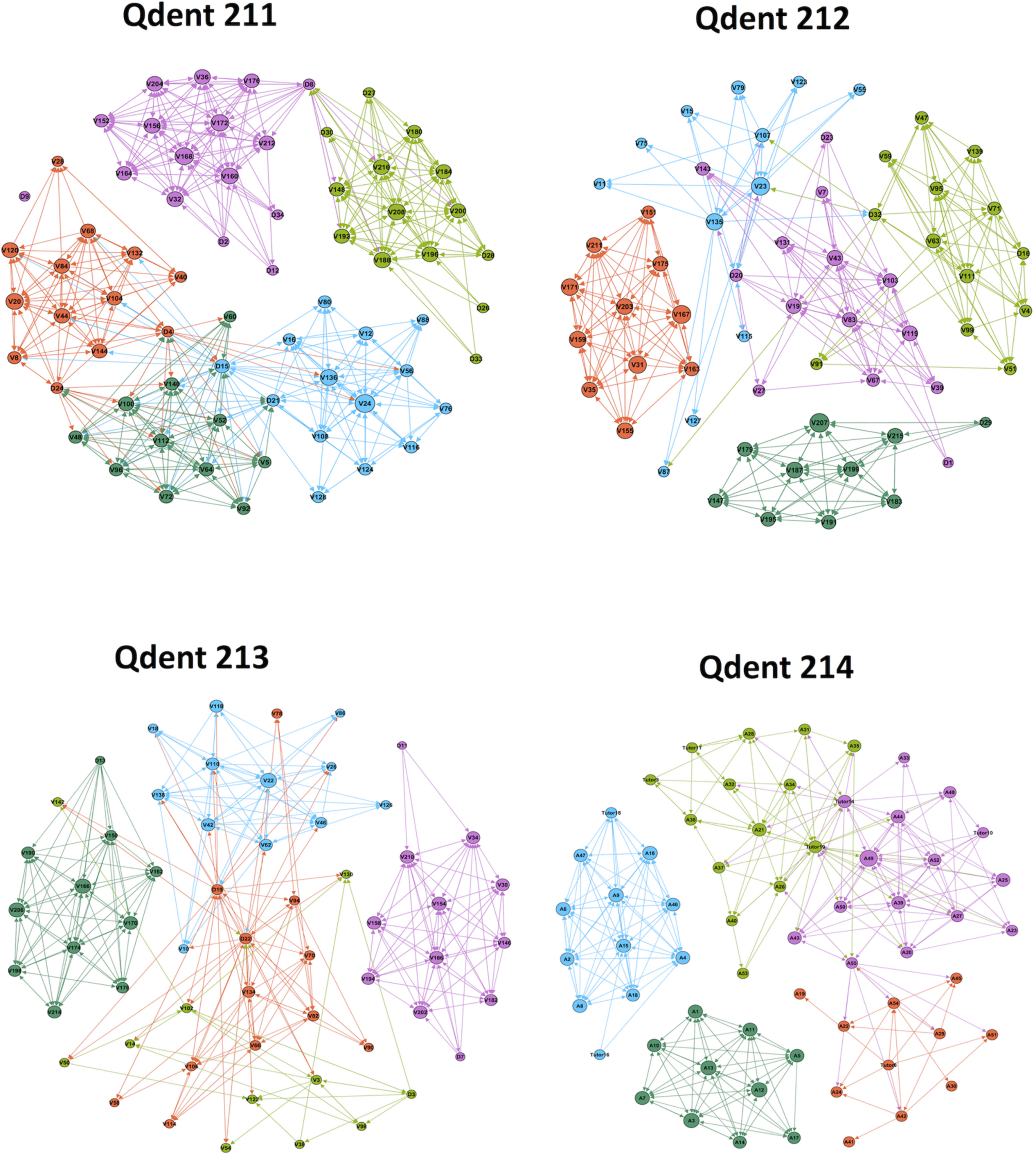
Summary of interactions in the four courses shows a bird eye view of courses and groups, level of interactivity and relations. Nodes (participants) are represented as circles, edges (interactions) are represented as arrows, and each circle size corresponds to the degree centrality (quantity of interactions), each group was given a unique color.

**Fig 3:**
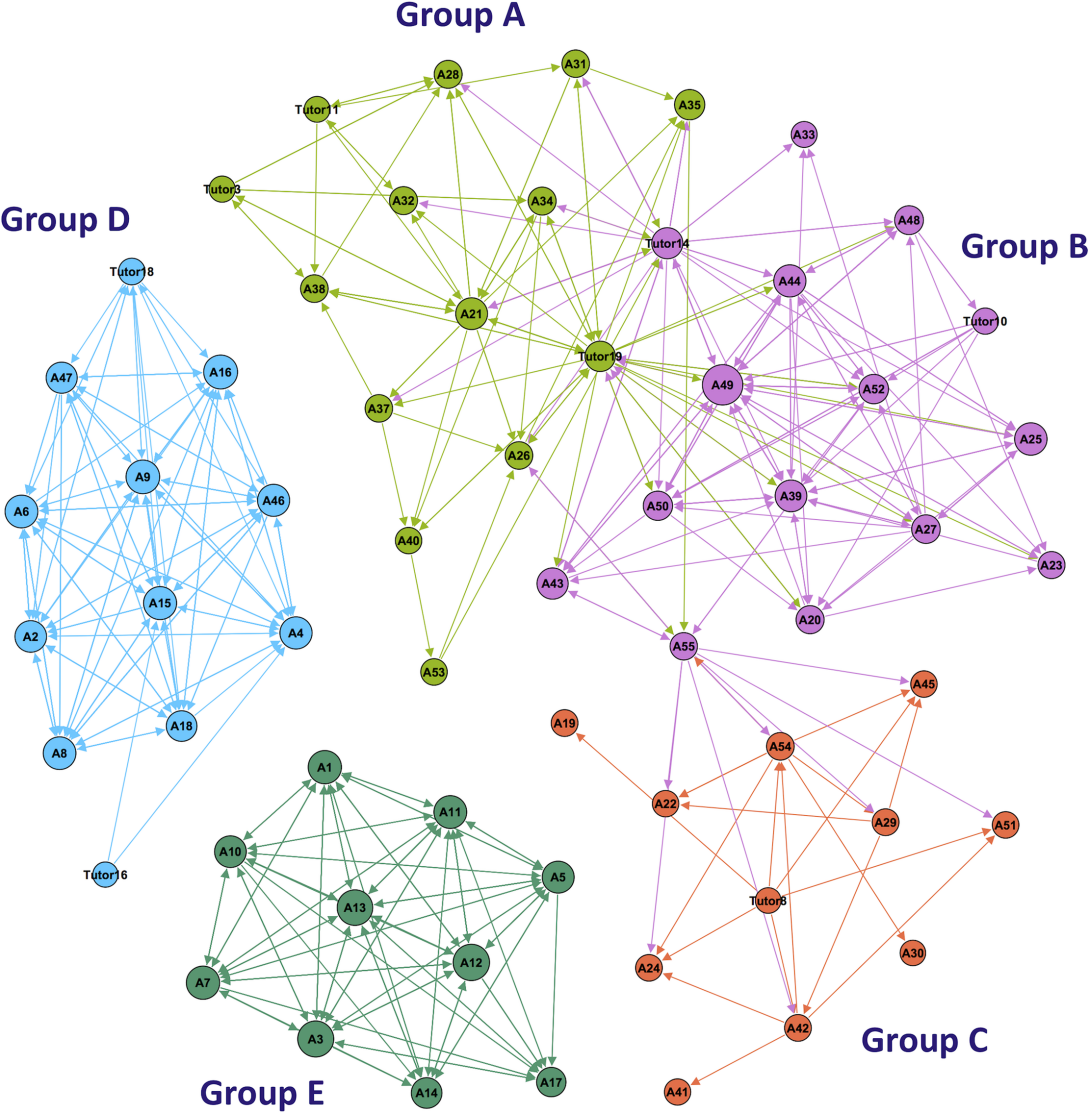
A closer view that summarizes all interactions in Principles of Dental Sciences course (QDENT 214), showing students and tutors activity levels and connectedness. Nodes (participants) are represented as circles, edges (interactions) are represented as arrows, and each circle size corresponds to the degree centrality (quantity of interactions), each group was given a unique color.

The visualization of course interactions presented in Fig 2 shows the four courses combined and in order to achieve a more detailed picture, we plotted the course “Principles of Dental Sciences” in Fig 3. Each group was assigned a unique color. The size of each node was configured to denote the *degree centrality*. Therefore, active/inactive students will have larger node sizes and can be visually recognized. The visualization outlines the interactions and relationships among participants in each course and provides an overview of the groups and their relation to each other. The level of interactivity in each group can be quickly assessed by the density of edges among nodes. Thus active and inactive groups can be quickly identified. An example in Fig 3 is group D and E, which shows marked interactivity, and group C, which was less interactive.

The network of each course—except for very infrequent bridges by the tutors—were divided into isolated components (the PBL groups). Because some of the centrality measures take into account the network size or path length, the centrality measures in our study were calculated for each group separately.

### RQ1: How do social network analysis indicators correlate to performance (in terms of grades) in online PBL?

To test what social network parameters might correlate with student’s performance, three groups of parameters were tested using Pearson’s correlation test. These were group properties, tutor, and student role. Table 3 shows the results of group and tutor role, and Table 4 shows student role. The results of the correlation test showed that the number of students in each group (group size) was negatively correlated with performance in all courses when the analysis was done per course basis and the overall results, and when the analysis was done using data of all students in all courses combined. Average group clustering coefficient (which measures group cohesion) as well as density (which measures group interactivity), followed by the measures of quantity of interactions (average degree, number of interactions and number of S-S interactions), were consistently moderate to strongly correlated with performance consistently in individual courses and in relation to the overall results.

Parameters corresponding to tutor role (average tutor degree, number of S-T interactions, number of T-S interactions) showed mixed results among courses, with either negative or statistically insignificant outcomes. Nonetheless, using data from all students, the tutor parameters correlation with performance were weakly and statistically insignificant. In summary, small and interactive cohesive groups with limited tutor role tended to perform better. Full details of results are listed in Table 3.

**Table 3:** Correlation between group network parameters and grades.

Parameter	QDENT 214 (N = 54)		QDENT 213 (N = 53)		QDENT 212 (N = 54)		QDENT 211 (N = 54)		Overall (215)	
	Correlation	P	Correlation	P	Correlation	P	Correlation	P	Correlation	P
Group size	-0.74	<0.01	-0.31	0.02	-0.63	<0.01	-0.48	<0.01	-0.57	<0.01
Group av. Degree	0.67	<0.01	0.68	<0.01	0.58	<0.01	0.60	<0.01	0.58	<0.01
Group density	0.73	<0.01	0.69	<0.01	0.63	<0.01	0.61	<0.01	0.62	<0.01
Group av. Clustering	0.71	<0.01	0.67	<0.01	0.66	<0.01	0.67	<0.01	0.73	<0.01
N. of interactions	0.53	<0.01	0.64	<0.01	0.53	<0.01	0.50	<0.01	0.53	<0.01
N. of S-S interactions	0.62	<0.01	0.66	<0.01	0.53	<0.01	0.47	<0.01	0.56	<0.01
N. of S-T interactions	-0.37	0.01	-0.68	<0.01	-0.33	0.01	-0.28	0.04	-0.06	0.37
N. of T-S interactions	-0.42	<0.01	-0.59	<0.01	-0.25	0.06	-0.34	0.01	0.02	0.77
Tutor degree	-0.44	<0.01	-0.69	<0.01	-0.56	<0.01	-0.20	0.16	-0.02	0.77

Av = Average, T = Tutor, S = student.

Three groups of parameters were investigated, the quantity of interactions, role in information transfer, and connectedness/social capital. Except for betweenness centrality, which showed mixed results in correlation with performance, there was a moderate to strong positive and statistically significant correlation with performance and student interaction indicators (quantity of participation, role in information exchange, connectedness and social capital parameters). The correlation was consistent -with slight variation in strength- in all courses and the results of all students combined. The correlation with the performance was highest in parameters measuring connectedness and social capital, namely in-degree, closeness centrality, prestige in-degree, prestige domain and prestige proximity. The detailed results are presented in Table 4, where the correlation between students’ network parameters and grades are shown.

**Table 4:** Correlation between students’ network parameters and grades.

Parameter	QDENT 214 (54)		QDENT 213 (53)		QDENT 212 (54)		QDENT 211 (54)		Overall (215)	
	Correlation	P	Correlation	P	Correlation	P	Correlation	P	Correlation	P
In-degree	0.66	<0.01	0.75	<0.01	0.64	<0.01	0.60	<0.01	0.51	<0.01
Out-degree	0.62	<0.01	0.66	<0.01	0.61	<0.01	0.57	<0.01	0.50	<0.01
Degree	0.65	<0.01	0.73	<0.01	0.65	<0.01	0.60	<0.01	0.51	<0.01
Closeness	0.64	<0.01	0.65	<0.01	0.69	<0.01	0.64	<0.01	0.53	<0.01
Betweenness	-0.15	0.28	0.19	0.18	0.12	0.38	0.10	0.46	0.16	<0.01
Eigen	0.52	<0.01	0.41	<0.01	0.36	0.01	0.59	<0.01	0.33	<0.01
Clustering	0.65	<0.01	0.58	<0.01	0.62	<0.01	0.64	<0.01	0.51	<0.01
Indegree prestige	0.64	<0.01	0.73	<0.01	0.61	<0.01	0.61	<0.01	0.53	<0.01
Domain prestige	0.40	<0.01	0.33	0.02	0.37	0.01	-	-	0.37	<0.01
Proximity prestige	0.70	<0.01	0.69	<0.01	0.61	<0.01	0.67	<0.01	0.54	<0.01
Rank prestige	0.71	<0.01	0.73	<0.01	0.59	<0.01	0.61	<0.01	0.47	<0.01
Eccentricity	-0.08	0.57	-0.32	0.02	-0.07	0.64	-0.42	<0.01	-0.24	<0.01

Av = Average, T = Tutor, S = student.

### RQ2: How far can SNA indicators be used as reliable predictors of performance in online PBL?

Two predictive models were performed, an explanatory model and a predictive model in order to predict performance:

#### 1. Explanatory model

An explanatory model is hypothesis driven. Three categories of factors may contribute to performance in PBL environment. These are the student, the tutor, and the group [3, 13, 14]. We included these three categories in a regression model to test how well they can predict performance. These parameters were group factors (group size, density of interactions, average previous GPA of other group members, and average clustering coefficient of other group members), tutor factors (tutor degree), student interactivity factors (in-degree, out-degree), role in information transfer (closeness centrality and betweenness centrality), social capital (Eigen centrality, prestige domain) in addition to demographic factors (age, gender, previous GPA).

A stepwise backward multivariable linear regression was done to test what SNA indicators may significantly explain variance in the final grade after controlling for previous performance, age, and gender. The adjusted R^2^ of the final model (5^th^ step) was 0.75, F (9,185) = 66.7, P<0.01). In addition to previous performance and female gender, the factors that reflected student interactivity such as density, clustering, and social capital were the most significant positive predictors of performance. In other words, a well-connected student in an interactive group where most members participate in the discussion is likely to score better. Whereas, the factors that reflect the strength of tutor role, large group size or a male gender were the negative predictors of performance. Full regression statistics are listed in Table 5.

**Table 5:** The significant predictors of grade using backward linear regression.

	Standardized beta Coefficients	t	P	Tolerance	VIF
Gender **	-0.64	-6.64	<0.01	0.14	7.32
Tutor out-degree *	- 0.14	- 2.30	0.02	0.33	3.07
Group size *	-0.12	-2.22	0.03	0.42	2.37
Eigen centrality **	0.13	3.08	<0.01	0.68	1.48
Density **	0.22	2.68	<0.01	0.20	5.07
Previous GPA **	0.51	9.23	<0.01	0.42	2.39
AV Group Clustering **	0.68	5.91	<0.01	0.10	10.0
Domain prestige	-0.11	-1.81	0.07	0.37	2.74
Closeness centrality	0.10	1.71	0.09	0.36	2.76

** Significant at the level of P<0.01.

* Significant at the level of P<0.05.

VIF = Variance inflation factor.

#### 2. Predictive model

The selection of predictors in a predictive model varies from an explanatory model, as it tries to include all information that can possibly add to the predictability [60, 61]. A stepwise backward logistic regression was performed to find how far using SNA indicators can successfully classify achievers and low-achievers. The -2 Log likelihood was 67.97, the Cox & Snell R Square was 0.6, and Nagelkerke R Square was 0.84 (Chi-square = 180.27, p < .001 with DF = 7). The Hosmer and Lemeshow goodness-of-fit test was (P = 0.28), indicating no evidence of poor fit. The model successfully classified 93.3% of cases, 88.24% of the low achievers, and 96.06% of the high achievers. The F-measure was 90%, and AUC was 0.92, full confusion matrix results are tabulated in Table 6. The Significant predictors were previous grade, Eigen centrality, density, and tutor out-degree; the full results are tabulated in Table 7.

**Table 6:** A confusion matrix of classified students using logistic regression.

	Observed Low	Observed High	Precision
Predicted Low	60	5	92.3%
Predicted High	8	122	93.8%
**Recall**	**88.2%**	**96.1%**	
Accuracy: 93.3%, F measure: 90%, AUC: 0.92			

**Table 7:** Predictors of achievement.

Parameter	Standardized Coefficient	Standard Error	P
Tutor Out-degree*	-0.087	0.037	0.021
Previous GPA**	0.487	0.103	<0.01
Density**	2.908	0.879	<0.01
Eigen centrality**	4.264	1.445	<0.01
AV Group Clustering	7.74	5.321	0.146
Gender	-5.705	2.936	0.052
group size	-0.669	0.455	0.141

** Significant at the level of P<0.01.

* Significant at the level of P<0.05.

### Validation

We used the study dataset as a training dataset and the next academic year as a testing dataset to examine how far the generated model can classify future students according to achievement. The testing dataset contained 183 students in the same four courses, using the model generated by the study dataset, we were able to correctly classify 82.7% of the underachievers in the testing dataset (next year) with an overall accuracy of 83.1% and F measure of 87.6%. The full confusion matrix is presented in detail in Table 8.

**Table 8:** A confusion matrix of classified students using the model developed by the training dataset.

Overall results (N = 183)			
	Observed Low	Observed High	Precision
Predicted Low	43	19	69.4%
Predicted High	12	109	90.1%
**Recall**	**78.2%**	**85.2%**	
**Accuracy: 83.1%, F-measure: 87.6%, AUC: 0.89**			

Applying the model on a course-wise basis, we were able to consistently predict the underachievers in each of the studied courses with reasonable precision and recall. In fact, the predictability (recall) improved to an average of 90.9% (range: 86.7%: 92.9%), F-measure ranged from 82.1% to 88.5%. It is clear that the model can be reliably used to classify under-achievers and high-achievers given the high recall of both categories. However, the model consistently identified some high achievers as potentially low achievers. The full details of each course confusion matrix and performance are presented in Table 9

**Table 9:** A confusion matrix of classified students using the model developed by the training dataset in each course separately.

**Body Systems in Health and Disease QDENT 214 (N = 47)**			
	Observed Low	Observed High	Precision
Predicted Low	**13**	5	72.2%
Predicted High	2	**27**	93.1%
**Recall**	**86.7%**	**84.4%**	
**Accuracy: 85.11%, F measure: 88.52%, AUC: 0.92**			
**General Surgery QDENT 213 (N = 47)**			
	Observed Low	Observed High	
Predicted Low	**13**	9	59.1%
Predicted High	1	**23**	95.8%
**Recall**	**92.9%**	**71.9%**	
**Accuracy: 78.26%, F-measure: 82.14%, AUC: 0.89.**			
**Principles of Dental Sciences QDENT 212 (N = 45)**			
	Observed Low	Observed High	
Predicted Low	**11**	9	55%
Predicted High	1	**24**	96%
**Recall**	**91.7%**	**72.7%**	
**Accuracy: 77.8%, F-measure: 82.8%, AUC: 0.85**			
**Neuroscience QDENT 211 (45)**			
	Observed Low	Observed High	
Predicted Low	**13**	6	68.4%
Predicted High	1	**25**	96.2%
**Recall**	**92.9%**	**80.7%**	
**Accuracy: 84.4%, F-measure: 87.7%, AUC: 0.93**			

## Discussion

The results of this study showed a consistent moderate to strong positive correlation between interaction parameters and performance across all the studied courses regardless of the subject matter. In each of the studied courses, students with stronger ties to prominent peers (better social capital) in small interactive and cohesive groups tended to perform better. The results of correlation tests were confirmed using regression tests, which were validated using a next year dataset.

To demonstrate the role SNA can play in capturing the relational construct and interaction parameters of online PBL, and possibly be used as predictors of performance, we created an explanatory regression model that included the factors commonly cited to affect performance in a PBL setting [3, 13, 14]. The model showed that a significant variance of grades could be explained by the group interactivity construct as measured by density of interactions, the cohesion of group members and the strength of students’ social ties, which emphasizes the role of social capital and interactivity as indicators of learning. The high accuracy obtained with the predictive model (93.3%) demonstrated the possibility of using interaction data to predict underachievers. Since predictive modeling is action-oriented, successfully identifying underachievers represents an obvious opportunity for intervention and allow for the provision of support before it is too late [49]. The usage of the next year dataset to validate the predictive potential of the obtained model adds to the credibility of the obtained results. The accuracy of identifying low achievers in the following year ranged from 86.7% to 92.9%, nevertheless with relatively low precision. A possible explanation might be due to the pattern of online activity of some high achieving students, who might participate online at levels indistinguishable from low achievers. Nonetheless, the issue that the algorithm identified most of the low achievers with high accuracy, and misclassified some of the high achievers as low achievers may be of less concern, and might be in favor of the students and educators alike. Casting a wide net is probably better than missing some underachievers [31].

Although results from correlation and linear regression tests seem to suggest a negative correlation between tutor interactions and students grades, they should not be viewed as contradicting research that has demonstrated a positive impact of knowledgeable and social congruent tutors [3, 14]. The tutor's parameters studied in this study are rather quantitative and correspond to the instances teachers helped students in inactive groups, and expectedly, tutors helped the less performing students more than they helped others.

While the early research results linking SNA to academic performance were promising, reproducing the obtained models on future iterations of these courses, have been either unsuccessful or untested [34–39]. Studies that investigated multiple courses have faced the same problem of reproducibility [30, 32, 62]. The difficulty to replicate results among studies and across different courses is an indication that the context in which the interactions occur has a significant role in the importance of different centrality measures and their predictive power [30, 36]. The results of this study have demonstrated that results can be consistent and reproducible from course to course and from year to year. The reason behind this consistency of research findings might be that the uniformity of the context, besides, the teaching method was similar in the studied courses, where the social interactions among learners and tutors in CSCL are the primary features of the learning process. Another reason may be due to carefully choosing predictors based on an established theoretical backdrop. Considerate selection of predictors improves prediction accuracy, speed, and enhances reproducibility [53, 61]. We tried in this study, to produce a set of predictors that are relevant to the context studied, more representative of students’ activities, can be interpreted on pedagogical grounds and offers better understanding of the underlying process and most importantly can be replicated by others trying to reproduce this results in similar contexts [44, 52, 53, 61].

We believe that another point of strength in this study lies in the modifiable predictors that were found to correlate with better learning. These modifiable factors can be improved and potentially improve the course outcome as the results of this research might indicate. Examples include enhancing course design to encourage interactivity and design problems that encourage constructive interactions [6, 18, 21, 63, 64]. It also includes helping isolated students with better access to social support in an inclusive environment that rewards collaborative learners [24, 34, 37, 38], and training tutors to be socially congruent, facilitators and supporters of an inclusive interdependent, collaborative learning process [3, 14].

In this study, SNA offered a wealth of information about students that were easy to obtain and interpret, in contrast to traditional content analysis methods that require effortful coding and time-consuming manual analysis that is impractical for monitoring online interactions on a large scale basis beyond research settings [65]. This is also true when comparing SNA to other research methods, such as observation or exploratory methods. SNA is a practical and cost-effective choice that is feasible to implement and can deliver timely effortless information about students, groups and the whole class. The insights offered can be automatically generated using learning management systems plugins [27, 28, 30, 66]. Two specific functions can offer insights, namely: 1) visualizations of online interactions and 2) learning analytics predictors that can be used to alert students who are not doing well and might be in need for support [25, 29, 36, 37].

A possible criticism for our approach is that adding more variables–particularly Non-SNA data- might have improved the predictive analytics model. However, we think that in this particular case, it might not be as intuitive as it seems. Two categories of data might be candidates for inclusion in our analysis, time-on-task and access data in the form of clicks and views. The first introduces a potentially inaccurate predictor, and the latest is strongly correlated with SNA quantitative data, albeit less relevant and noisy (introduces bias, interdependence and decrease the prediction performance). Time recording tools are mostly inaccurate, produce mixed results, and poses a threat to the quest for replicable and reproducible research in analytics [67, 68]. Judd, 2014 [67] used special tracking devices to record student’s online activities to investigate the multitasking behavior; they found that multitasking was significantly present in 99% of the recorded sessions, acting as a serious confounding of the time-on-task [67]. Kovanović et al., 2015 [68] studied the influence of fifteen different time-on-task measurement techniques on model learning analytics performance. They concluded that based on the challenges in accurate estimation of time-on-task and the absence of clear methodologically standardized estimation strategy, the inclusion of time-on-task in learning analytics models should be re-considered for the sake of clear, sound and replicable data analysis strategies [68]. The other set of predictors are the parameters derived from students’ logs such as number of logins, clicks on resources, and views. While these predictors might seem relevant, they are strongly correlated and interdependent with the quantitative SNA parameters. Both SNA quantitative measures and these measures do essentially measure the same thing; the difference is that SNA quantitative measures reflect access to the resources that are more relevant to the program and less susceptible to have noise [53, 61].

Since online learning is a vast and rather diverse field, the results of this study remain to be tested in other interactive course environments. Our results might have contextual constraints that might limit the generalizability into other contexts.

## Conclusions

The findings of this study have shed light on the role of interactivity and the relational construct in the online PBL process, by means of a novel technique. Using Social Network Analysis to study online interactions has offered insights that help us to predict under-achievers and uncover the significance of the role of learner-learner and learner-tutor interactions in relation to performance.

Our results showed a consistent moderate to strong positive correlation between performance, interaction parameters and students’ centrality measures across all the studied courses, regardless of the subject matter. In each of the studied courses, students with stronger ties to prominent peers (better social capital) in small interactive and cohesive groups tended to do better. The results of correlation tests were confirmed using regression tests, which were validated using a next year dataset. Using SNA indicators, we were able to classify students according to achievement with high accuracy (93.3%). This demonstrates the possibility of using interaction data to predict underachievers with reasonable reliability, which is an obvious opportunity for intervention and support.
